# Absorbable Sutures are Equally Efficacious to Non-absorbable Sutures in Upper Eyelid Blepharoplasty for Dermatochalasis: A Comparative Study

**DOI:** 10.1007/s00266-025-05286-w

**Published:** 2025-10-06

**Authors:** Salvatore Giordano, Davide De Vita, Andre’ Salval, Pietro G. Di Summa, Carlo M. Oranges

**Affiliations:** 1 Department of Surgery, Satasairaala Hospital, 28500 Pori, Finland; 2 The University of Turku, 20014 Turku, Finland; 3 Department of Surgery, Welfare District of Forssa, 30100 Forssa, Finland; 4 Department of Plastic and Hand Surgery, University Hospital of Lausanne (CHUV), University of Lausanne, Lausanne, Switzerland; 5 Department of Plastic, Reconstructive and Aesthetic Surgery, Geneva University Hospitals, University of Geneva, 1201 Geneva, Switzerland

**Keywords:** Dermatochalasis, Blepharoplasty, Upper eyelid, Suture, Absorbable suture, Complications

## Abstract

**Background:**

Upper blepharoplasty for dermatochalasis is a common oculoplastic procedure worldwide, but postoperative suture removal can be uncomfortable or impractical for some patients.

**Aims and Objectives:**

This study aimed to compare outcomes between absorbable and non-absorbable sutures used for upper eyelid blepharoplasty closure and to assess differences in complication rates.

**Materials and Methods:**

We analyzed data from consecutive upper eyelid blepharoplasty procedures performed over a 3 year period. Patients were categorized based on the type of suture used for skin closure, with exclusion criteria including any prior eyelid or orbital surgery. Postoperative complications, outcomes, and aesthetic satisfaction scores (1–10) were compared between the two suture groups.

**Results:**

The study included 149 patients: 69 with absorbable sutures and 80 with non-absorbable polypropylene sutures. Demographic variables were similar between groups. Operative time, estimated blood loss, return to work and follow-up duration were comparable. No significant differences in complication rates were observed (2.9% vs. 2.5%, *p  *=  1.000), though a slight trend toward reoperation was noted in the absorbable suture group (8.7% vs. 6.2%, *p *= 0.570). Patient and surgeon satisfaction scores were similar.

**Conclusion:**

Our findings suggest that absorbable sutures are a viable and safe alternative to non-absorbable sutures for upper eyelid blepharoplasty, with no significant differences in postoperative outcomes or overall satisfaction.

**Level of Evidence III:**

This journal requires that authors assign a level of evidence to each article. For a full description of these Evidence-Based Medicine ratings, please refer to the Table of Contents or the online Instructions to Authors www.springer.com/00266.

## Introduction

Aging leads to a reduction in skin elasticity and thinning of the periorbital epidermis, which results in skin laxity. This senile degeneration can manifest in various ways, including sagging and drooping eyelids, multiple folds in the upper eyelids, drooping eyebrows, and ptosis [1]. These changes in the periorbital skin contribute to an aged appearance and can also cause issues such as visual field defects, entropion, trichiasis, and epiphora. The sagging skin can extend past the upper eyelid margin, sometimes even partially covering the palpebral fissure. Such changes not only impact cosmetic appearance but can also obscure the pupil, potentially affecting vision [2]. Re-laxation of the orbital septum and fat can exacerbate sagging, resulting in a fatigued appearance. This can cause a patient to appear listless and may even impair vision [3]. Consequently, correcting the aging upper eyelid is not only pursued for cosmetic reasons but also to improve upper eyelid function.

Recently, surgical procedures aiming to address eyelid aging have become more common. Upper blepharoplasty is one of the most frequently performed plastic surgery procedures worldwide [4]. Its popularity stems from its ability to produce quick and satisfactory results, often being conducted as an outpatient procedure under local anesthesia.

To close upper blepharoplasty incisions, fine non-absorbable sutures are typically used due to their ease of tying, durability, and minimal inflammatory response [5] in intradermal or in running continuous fashion. However, removing these sutures, even using topical local anaesthetic ointment can be time-consuming, negatively affecting patients’ experience and increase the costs. In contrast, rapid absorbable sutures do not require removal, do not disturb the scar, save time for the surgeon and nurses, and reduce patient anxiety, while improving patients’ satisfaction. The use of absorbable sutures may be particularly advantageous for elderly patients who face challenges with postoperative suture removal or for those who prefer this option. Nevertheless, most surgeons favor non-absorbable sutures, as they are perceived to better limit inflammation and reduce the risk of local complications, such as suture rejection.

Although absorbable sutures have been shown to be beneficial for skin closure in facial tumor surgeries, their use for eyelid incisions in upper blepharoplasty has not been extensively evaluated. The available studies on this topic are limited and primarily focus on younger patients or procedures performed for aesthetic purposes [6–9]. The only randomized study to date included only 36 patients [8]. However, the choice of suture material may influence wound healing, with a higher rate of wound dehiscence reported when using fast-absorbing plain gut sutures compared to polypropylene sutures [9].

The objective of this study was to compare the use of absorbable versus non-absorbable sutures in patients undergoing upper blepharoplasty for dermatochalasis, particularly in terms of the risk of postoperative complications, as well as patient and surgeon satisfaction.

## Materials and Methods

This is a retrospective cohort study from a prospectively maintained database and includes 149 patients diagnosed with symptomatic dermatochalasis. The cohort consists of consecutive patients who underwent upper blepharoplasty over a 3 year period. The study adhered to the ethical principles outlined in the World Medical Association Declaration of Helsinki and received approval from the local Institutional Review board. Individual informed consent was waived due to the retrospective nature of the data and its de-identification.

The patients’ demographic data were collected meticulously from the electronic medical records including preintervention reviews of medical and ophthalmologic histories, and follow-up outpatient clinics.

Inclusion criteria were as follows, diagnosis of dermatochalasis, including:

Upper eyelid skin redundancy causing a clear obstruction to vision (significant visual field narrowing), such as a lateral skin fold that blocks peripheral vision.

Eyelashes or skin rubbing against the eye or cornea.

Documented recurrent eyelid skin abrasions and infections despite appropriate care due to skin laxity.

Exclusion criteria included: previous upper eyelid procedures, concurrent procedures performed alongside upper blepharoplasty, pregnancy, age below 18 or above 100 years. Presence of any dermatological condition affecting the eyelid or face before the procedure and follow-up less than 3 months.

No additional surgical procedures for ptosis correction, such as levator advancement or resection, were performed during blepharoplasty. All cases of ptosis included were mild, involutional in nature, and did not result in significant functional impairment. Therefore, the surgical approach was limited to aesthetic upper eyelid contouring without addressing the ptosis directly.

Patients on antiplatelet or anticoagulant medications, such as acetylsalicylic acid, rivaroxaban, clopidogrel, warfarin, or other blood thinners, were advised to discontinue these medications 3–5 days before surgery. This was done under the guidance of the patient’s primary care physician and in accordance with local institutional guidelines.

The study population reflects the general demographic characteristics of Finland, primarily comprising Western populations, particularly Finnish patients with symptomatic dermatochalasis. This cohort represents the diverse range of patients commonly seen in Finland’s public healthcare system, which may affect the generalizability and real-world applicability of the study’s findings. Over 80% of the study population had Fitzpatrick skin types I–III [10].

Patients were monitored through physical examinations at follow-up visits scheduled for 15 days, 3–6 months, and at the last follow-up after the surgical procedure.

For the purpose of this study, patients were divided into two groups based on the suture material used. In Group A (absorbable suture group) was used a running intracutaneous 6-0 absorbable poliglecaprone 25 suture (Monocryl; Ethicon, Inc., Somerville, NJ). In Group B (non-absorbable suture group), a subcuticular intracutaneous 6-0 non-absorbable polypropylene suture (Prolene 6/0; Ethicon Inc., Somerville, NJ) was employed. The decision regarding which suture to use was at the discretion of the operating surgeon, based on patient logistics and preferences.

Group A consisted of 69 patients, while Group B had 80 patients. All patients underwent upper eyelid blepharoplasty using a similar technique employed by the author under local anesthesia. A number 15C blade was used for all skin incisions. Efforts were made to maintain consistency in incision length, handling of eyelid skin and soft tissue, and surgery duration.

Before the operation, patients were marked while standing, following a skin pinch test to assess excess skin for removal. The decision to remove upper eyelid fat was made in consultation with the patients, based on the eyelid’s appearance in various gaze positions (lateral, medial, upper, and lower).

Anesthesia was administered prior to surgery, typically with 5 ml of 1% lidocaine with 1:100,000 adrenaline per eyelid, using a 5 ml luer-lock syringe. Each eyelid was operated on approximately 20 min after the local anesthesia was injected. Excess skin, marked preoperatively in the upright position, was excised using a number 15C blade; the lateral part of the orbicularis muscle was then resected if deemed using needlepoint monopolar radio-surgery cautery (RadioSurg 2200, Aurum Medical, Moscow, RU). Finally, the septum was opened, and nasal and herniated excessive fat pads were reduced as needed. After achieving accurate hemostasis with bipolar cautery, the septum was closed with a single 5/0 Vicryl Rapid stitch (Ethicon, Raritan, NJ), and the skin was sutured according to the protocol for Group A or Group B. Blood loss was estimated by weighing the gauzes used during the procedures.

After the procedure, Steri-Strips (3M, Saint Paul, MN) and cool pads were applied in both groups A and B. All patients were advised to use cool pads five times a day for 20 min each time during the first 5 days. They were also advised to avoid heat sources and to sleep in a slightly elevated position with their head. Return to work was assessed by the duration of sick leave (in days) prescribed for patients who were not retired yet.

Sutures for Group B were removed after 10–14 days by a nurse. Post-surgery, patients were instructed to avoid any sports activities and direct sun exposure or other heat sources for 4–6 weeks.

The primary outcome measure was the occurrence of any surgical site occurrence (SSO), including wound dehiscence, incisional erythema, wound infections, and unacceptable scarring (such as hypertrophic scarring and uneven scars). Additionally, evaluations were made for persistent lagophthalmos, ptosis, xerophthalmia, and other upper eyelid anomalies.

Secondary outcomes included other complications and revision rates at the last follow-up. Indeed, each patient was verbally surveyed about their satisfaction using a visual analog scale (VAS) ranging from 1 (worst) to 10 (excellent) at the final postoperative follow-up, conducted by the surgeon who performed the blepharoplasty. Additionally, the surgeon recorded his overall satisfaction with the procedure’s outcomes.

Statistical analysis was performed using SPSS software (IBM SPSS Statistics, version 29, Armonk, NY, USA). Continuous variables were summarized with mean and standard deviation. Normality was assessed through histograms, Kurtosis, Skewness, and occasionally using the Kolmogorov–Smirnov test. For univariate analysis and comparisons between the two study groups based on suture material, Pearson’s Chi-square test, Fisher’s exact test, and t tests were utilized as appropriate. A two-sided *p* value of less than 0.05 was deemed statistically significant. A sample size calculation was performed to determine the number of participants required to detect a significant difference in surgical site outcomes (SSO) with 80% power and a significance level of 0.05, and Cohen’s d post hoc analysis was also performed for patient’s satisfaction.

## Results

A total of 149 patients were included in the study, and demographics were similar for all the variables considered (Table 1). Of these, 69 cases involved the use of absorbable sutures for skin closure, while 80 cases employed a running-locking cutaneous or intracutaneous non-absorbable polypropylene suture. The mean age of the patients was similar between the two groups, with the absorbable suture group having an average age of 72.5 years and the non-absorbable suture group having an average age of 65.1 years (*p *= 0.109).

**Table 1:** Demographics of patients at time of study

	Group A (*n* = 69)	Group B (*n* = 80)	*p*-value
Age (mean ± SD)	72.5 ± 8.8	65.1 ± 9.9	0.109
BMI^a^	27.3 ± 8.5	27.5 ± 5.9	0.768
Any comorbidity	37 (56.1%)	51 (63.7%)	0.210
HTA^b^	32 (46.8.0%)	45 (56.2%)	0.229
Diabetes	8 (11.9%)	13 (16.2%)	0.415
Hypercholesterolemia	23 (36.8%)	29 (36.2%)	0.710
Lung disease	12 (17.9%)	11 (13.7%)	0.540
Depression	6 (9.0%)	9 (11.2%)	0.605
Smokers	11 (16.4%)	12 (15.0%)	0.874
Ptosis	6 (9.2%)	3 (3.7%)	0.206
Asymmetry	24 (36.4%)	23 (28.7%)	0.429

^a^BMI, body mass index expressed in kg/m^2^ ^b^HTA, arterial hypertension

In terms of intraoperative and postoperative measures, we found that the operative time was comparable between the two groups. Similarly, the estimated blood loss during surgery was consistent across both groups (Table 2). Additionally, the time required for patients to return to work (for those who were employed) and the length of follow-up periods were also similar between the two groups. No differences were observed in the extent of orbicularis muscle or fat resection between the groups. Follow-ups were similar among the two groups; both were over one year (Table 2).

**Table 2:** Comparison of perioperative parameters in the two groups of patients

	Group A (*n* = 69)	Group B (*n* = 80)	*p*-value
Operative time (min, mean ± SD)	65.8 ± 18.8	63.7 ± 16.6	0.493
Estimated blood loss (ml, mean ± SD)	6.6 ± 5.3	6.4 ± 4.1	0.849
Orbicularis resection	11 (15.9%)	18 (22.5%)	0.313
Fat resection	10 (17.4%)	12 (12.5%)	0.930
Return to work (days, mean ± SD)*	8.3 ± 4.3	8.8 ± 3.6	0.631
Follow-up (months, mean ± SD)	14.3 ± 29.7	18.9 ± 18.7	0.083

*For patients not retired (6 vs. 38 patients)

Regarding postoperative complications, there were no significant differences in the overall incidence of complications between the two groups, with a rate of 2.9% in the absorbable suture group and 2.5% in the non-absorbable suture group (*p *= 1.000, Table 3). Two cases of postoperative ptosis were observed (Table 3); however, no intraoperative levator injury was noted in either case. The ptosis in both patients was mild and transient, resolving spontaneously within 3 months without the need for further surgical intervention. These findings suggest a temporary dysfunction rather than structural damage. There was a slight trend toward an increased rate of re-operation during the follow-up in patients where absorbable sutures were used (8.7% vs. 6.2%, *p *= 0.570). The re-operations were primarily due to postoperative asymmetry or insufficient skin resection.

**Table 3:** Postoperative complications and satisfaction

	Group A (*n* = 69)	Group B (*n* = 80)	*P*-value
Complications (SSO)*	2 (2.9%)	2 (2.5%)	1.000
Hematoma (requiring any procedure)	0 (0.0%)	0 (0.0%)	1.000
Any infection	1 (1.5%)	1 (1.2%)	1.000
Wound dehiscence (requiring any intervention or extra observation)	0 (0.0%)	0 (0.0%)	1.000
Ecchymosis (requiring any extra observation)	0 (0.0%)	0 (0.0%)	1.000
Postoperative ptosis	1 (1.5%)	1 (0.3%)	0.313
Levator damage	0 (0.0%)	0 (0.0%)	1.000
Re-operation	6 (8.7%)	5 (6.2%)	0.570
Patients’ satisfaction (0–10, mean ± SD)	7.6 ± 2.6	7.8 ± 2.6	0.840
Surgeon’s satisfaction (0–10, mean ± SD)	8.2 ± 2.3	8.0 ± 2.5	0.874

*SSO, surgical site occurrence

Both subjective patient and surgeon satisfaction levels were similar across the groups (Table 3). A sample size calculation based on our outcomes indicated that 1416 patients would be required to possibly detect a significant difference in surgical site outcomes (SSO) with 80% power. Cohen’d for patient’s satisfaction was 0.08, showing a small effect size.

## Discussion

This study aimed to compare the outcomes of two different suture materials in skin closure: absorbable sutures versus non-absorbable polypropylene sutures, in a cohort of 149 patients. The analysis revealed that the two groups were well-matched in terms of baseline characteristics considered for this study, such as age and comorbidities, ensuring that the comparisons made are robust and free from a significant selection bias.

Previous studies on this subject have reported similar outcomes, albeit with slightly different suture techniques [6–9]. Similarly to our findings, Jaggi et al. [6] observed no significant differences between absorbable and non-absorbable sutures in terms of scar quality, pain, and overall satisfaction, studying only 28 patients. Joshi et al. [7] concluded that using two interrupted 6-0 Prolene sutures along with a running 6-0 fast-absorbing gut resulted in the lowest rates of complications and revisions, though their follow-up period was much shorter, lasting only up to 3 months postoperatively, although in a prospective nonrandomized design and enrolling 800 consecutive patients. Kouba et al. [8] in their randomized, split-eyed, single-blind, controlled trial observed that cosmesis using epidermal closure and ethylcyanoacrylate was superior to fast-absorbing gut, including 36 consecutive blepharoplasties with up to 3-month follow-up.

Homer et al. [9] performed the largest study on this topic including a total of 2376 cases and they found that wound dehiscence occurrence was associated with male gender and fast-absorbing plain gut suture, with up to 2-month follow-up. In our study, which is the third largest on this topic, we did not find such differences, having a much longer follow-up. However, we used Monocryl in the experimental group, which retains 25% of its tensile strength at 14 days, and it is completely resorbed at 90–120 days according to Ethicon manufactory (Monocryl; Ethicon, Inc., Somerville, NJ). This fact can explain our extremely low wound dehiscence. Nevertheless, compared to previous studies, our cohort had a higher mean age (69 years) than those reported in other studies, which ranged from 52 years [6], 55 years [8], to 67 years [9]. This highlights the clinical and functional significance of the procedures, particularly in an older population with high prevalence of comorbidities (Table 1). Nowadays, our institution has adopted the use of absorbing suture for the closure of upper eyelid blepharoplasties in elderly patients or whenever requested.

No differences were observed in the extent of orbicularis muscle or fat resection between the groups. This suggests that the surgical procedures were performed with a similar approach and consistency, regardless of the suture material, ensuring that the surgical techniques themselves were consistent across the study population (Table 2).

Our findings showed that operative time, estimated blood loss, return to work, and follow-up durations were remarkably similar between the two groups. This indicates that the choice of suture material—whether absorbable or non-absorbable—did not significantly affect these key surgical measures, a fact not previously fully reported in previous studies on this topic.

These results are consistent with previous research in oculoplasty procedures, which has shown that suture material generally has minimal impact on operative time and immediate postoperative recovery metrics, if the suturing technique is appropriate for the procedure and tissue type [6–12].

Importantly, there were no significant differences in the incidence of complications between the two groups (2.9% vs. 2.5%). The occurrence of postoperative ptosis in the absence of documented levator injury may be attributed to transient factors such as postoperative edema or tension from the suture line affecting levator function. Given the spontaneous resolution in both cases, it is unlikely that permanent muscle damage occurred. The overall low complication rate is encouraging and suggests that both suture types are safe and effective for skin closure in this patient population. However, the slight trend toward a higher re-operation rate in the absorbable suture group (8.7% vs. 6.2%) may warrant further consideration. Although this difference was not statistically significant, it may hint at a potential draw-back of absorbable sutures in certain patients.

These outcomes were similar to the previous four studies on this topic [6–9] reporting 2.5–5.5% of complications.

In our cohort, the primary reasons for re-operation were postoperative asymmetry and insufficient skin resection. These findings underscore the importance of precise surgical technique and patient selection. While the suture material itself may not be the direct cause of these issues, the gradual loss of tensile strength in absorbable sutures might contribute to a higher likelihood of tissue laxity over time, potentially necessitating further intervention. Additionally, the presence of postoperative asymmetry as a cause for re-operation suggests that suture material, combined with the surgeon’s technique and the patient’s individual healing process, play a critical role in the aesthetic outcomes of surgery. Future studies should explore whether certain patient populations, such as those with compromised wound healing or higher skin laxity, are more prone to these complications when absorbable sutures are used.

Patients’ satisfaction was not higher in group A, although there was no need for suture removal. Patient and surgeon satisfaction was similar across both groups, indicating that both suture types are generally well accepted and do not significantly impact on perceived outcomes. This aligns with the notion that patient satisfaction is multifactorial, influenced by overall surgical results, postoperative care, and individual patient expectations, rather than solely by the type of suture used. High satisfaction is generally reported after upper eyelid blepharoplasty [11, 12]. It is also worth noting that satisfaction levels are likely tied to the lack of significant complications and the overall success of the procedures. A recent report suggests that treating any degree of dermatochalasis, even without functional impairments, may be necessary to achieve patient satisfaction with the cosmetic results. Additionally, the functional benefits and improvements in the visual field highlight the procedure’s impact beyond just its aesthetic aspects [13]. This indicates that if the primary outcomes of surgery, such as effective wound closure and aesthetic results, are achieved, the type of suture material may not be a major determinant of patient or surgeon satisfaction.

These results showed that both suture materials can be effectively used for skin closure, with the choice depending on surgeon preference, patient factors, and specific clinical scenarios. For instance, in patients where long-term tensile strength is crucial, non-absorbable sutures may be favored to reduce the risk of re-operation. Conversely, in cases where suture removal might pose a challenge, absorbable sutures offer a convenient alternative without compromising safety or satisfaction, providing important cost savings, particularly in public healthcare settings.

It is also essential to consider the patient’s overall health and risk factors when selecting the suture material. For example, patients with chronic conditions such as diabetes, which may impair wound healing, might benefit from non-absorbable sutures that provide prolonged support, reducing the risk of wound dehiscence. We could not analyze this issue, as there were no cases of wound dehiscence in our cohort (Table 3). Similarly, antibiotic-coated sutures as well as tissue sealants might be used to further optimize the outcomes in diabetic patients and/or patients taking antiplatelet medications [14, 15]. Furthermore, in cosmetic surgeries where aesthetic outcomes are paramount, the choice of suture material should be carefully considered to balance ease of use, potential for scarring, and long-term results [16, 17].

This is the third largest study on this topic [6–9] including a long follow-up (over one year) and consistent study population. The primary value of this study lies in its exploration of absorbable sutures as a viable option for elderly patients, a demographic well represented in our cohort. This is a significant strength, as it provides insights that may not have been fully addressed in previous research, which primarily included younger populations. This is particularly important for elderly patients, who often face challenges with postoperative care, such as suture removal, thereby enhancing the generalizability and clinical relevance of our findings. Our findings support the conclusion that both types of sutures facilitate efficient and effective surgical workflows without adding additional burdens in terms of operative time or patient recovery. We believe that absorbable sutures can improve patient’s comfort and cost reduction because it is not needed additional appointment at the clinic for suture removal.

The major limitations of this study include its retrospective nature and relatively small sample size, particularly given the prospective change in suture material, which may limit the generalizability of the findings. The study population reflects a demographic typical of Finland and primarily represents Western populations, with a high prevalence of individuals with lower Fitzpatrick skin types. Finland’s publicly funded healthcare system influences access to procedures like blepharoplasty, particularly in terms of cost, patient demographics, and cultural factors, with surgeries often performed for functional rather than purely cosmetic reasons. These factors may limit the generalizability of the findings to countries with different healthcare models, demographics, or cultural norms. Additionally, the follow-up period may not have been sufficient to capture late complications that could arise from either suture type or scarring issues. The lack of randomization also introduces potential bias, as the choice of suture material might have been influenced by specific patient or surgical factors that were not fully accounted for. Furthermore, we did not assess scar quality or pain, as previously reported [6, 7, 18]. However, operated patients were instructed to contact the clinic in case of any issues after the last control. Another important limitation of this study is the absence of validated patient-reported outcome measures (PROMs), such as the FACE-Q Eye Module or the Blepharoplasty Outcomes Evaluation (BOE) Questionnaire. Instead, a visual analog scale (VAS) was used, reflecting its routine use and ease of implementation in our clinical setting at the time of data collection. While this approach provided practical insight, it may lack the psychometric robustness of validated tools. Additionally, surgeon-reported outcomes were based on clinical examination and photographic review rather than a standardized grading system. Similarly, another limitation of this study is the lack of assessment for scar quality and postoperative pain using validated evaluation tools. Although these are important aesthetic and patient-centered outcomes in blepharoplasty, they were not measured using standardized scales such as the Patient and Observer Scar Assessment Scale (POSAS) or the Vancouver Scar Scale (VSS), primarily due to the retrospective design and absence of such tools in routine clinical practice at the time. While standardized photographs were obtained for clinical documentation, these images were not assessed by blinded evaluators, which may introduce observer bias. These factors should be considered when interpreting the comprehensiveness of the outcome evaluation.

Future research should aim to conduct larger, prospective studies with longer follow-up durations to validate these findings and explore the long-term outcomes of different suture materials, including validated patient-reported outcome measures [13]. Additionally, the inclusion of more diverse patient populations across various surgical disciplines would help in generalizing the results and understanding the broader applicability of these findings.

## Conclusions

This study demonstrates that both absorbable and non-absorbable sutures are viable options for skin closure in upper eyelid blepharoplasty, with no significant differences in major surgical outcomes or complication rates. These findings contribute to the ongoing discussion on optimizing surgical techniques and materials to enhance patient outcomes. Future studies should further investigate the potential long-term benefits and drawbacks of each suture type, with a focus on specific patient populations and surgical contexts, to provide clearer guidelines for clinical practice.

## References

[1] Lee TY, Shin YH, Lee JG. Strategies of upper blepharoplasty in aging patients with involutional ptosis. Arch Plast Surg. 2020;47(4):290–6. 10.5999/aps.2020.01361. 32718105 10.5999/aps.2020.01361 PMC7398815

[2] Xu P, Huang H, Zhang S, Yin X, Zhang Q, Du Y. A comprehensive approach to upper eyelid rejuvenation surgery. Aesthetic Plast Surg. 2021;45(3):1047–55. 10.1007/s00266-020-02031-3. 33403413 10.1007/s00266-020-02031-3

[3] Gülbitti HA, Bouman TK, Marten TJ, van der Lei B. The orbital oval balance principle: a morphometric clinical analysis. Plast Reconstr Surg. 2018;142(4):451e–61e. 10.1097/PRS.0000000000004805. 29979364 10.1097/PRS.0000000000004805

[4] Hicks K, Sclafani AP, Thomas JR. Evolution of blepharoplasty. Facial Plast Surg. 2019;35(4):340–52. 10.1055/s-0039-1693437. 31470462 10.1055/s-0039-1693437

[5] International Society of Aesthetic Plastic Surgeons. Global survey 2023: full report and press releases (English). 2024. https://www.isaps.org/media/rxnfqibn/isaps-global-survey_2023.pdf. Accessed Aug 4 2024.

[6] Joshi AS, Janjanin S, Tanna N, Geist C, Lindsey WH. Does suture material and technique really matter? Lessons learned from 800 consecutive blepharoplasties. Laryngoscope. 2007;117(6):981–4. 10.1097/MLG.0b013e31804f54bd. 17545862 10.1097/MLG.0b013e31804f54bd

[7] Jaggi R, Hart R, Taylor SM. Absorbable suture compared with nonabsorbable suture in upper eyelid blepharoplasty closure. Arch Facial Plast Surg. 2009;11(5):349–52. 10.1001/archfacial.2009.53. 19797100 10.1001/archfacial.2009.53

[8] Kouba DJ, Tierney E, Mahmoud BH, Woo D. Optimizing closure materials for upper lid blepharoplasty: a randomized, controlled trial. Dermatol Surg. 2011;37(1):19–30. 10.1111/j.1524-4725.2010.01834.x. 21199097 10.1111/j.1524-4725.2010.01834.x

[9] Homer NA, Zhou S, Watson AH, Durairaj VD, Nakra T. Wound dehiscence following upper blepharoplasty: a review of 2,376 cases. Ophthalmic Plast Reconstr Surg. 2021;37(3S):S66–9. 10.1097/IOP.0000000000001816. 32852369 10.1097/IOP.0000000000001816

[10] Sinikumpu SP, Jokelainen J, Keinänen-Kiukaanniemi S, Huilaja L. Skin cancers and their risk factors in older persons: a population-based study. BMC Geriatr. 2022;22(1):269. 10.1186/s12877-022-02964-1. 35361154 10.1186/s12877-022-02964-1 PMC8973875

[11] Manasseh GSL, Hunt SV, Garrott H, Ford RL, Caesar R, Harrad RA. Anterior approach ptosis surgery: comparison of absorbable polyglactin sutures and non-absorbable polyester sutures. Does Vicryl stand the test of time? Orbit. 2022;41(5):547–50. 10.1080/01676830.2021.1958873. 34334084 10.1080/01676830.2021.1958873

[12] Moledina M, Ahmed I, Ranji A, Chipeta C, Caesar R, Malik A. Lateral tarsal strip procedure: comparison of absorbable sutures and non-absorbable polypropylene suture. Does the suture type matter? Eye (Lond). 2024;38(4):752–6. 10.1038/s41433-023-02768-6. 37857715 10.1038/s41433-023-02768-6 PMC10920783

[13] Marangi GF, Mirra C, Savani L, Cogliandro A, Romano FD, Segreto F, et al. Is the severity of preoperative eyelid dermatochalasis directly correlated with patient satisfaction after upper blepharoplasty? a prospective study based on PROMs. Aesthet Plast Surg. 2024. 10.1007/s00266-024-04214-8. 10.1007/s00266-024-04214-8 39037480

[14] Biancari F, Giordano S. Glycated hemoglobin and the risk of sternal wound infection after adult cardiac surgery: a systematic review and meta-analysis. Semin Thorac Cardiovasc Surg. 2019;31(3):465–7. 10.1053/j.semtcvs.2019.02.029. 30851374 10.1053/j.semtcvs.2019.02.029

[15] Giordano S, Koskivuo I, Suominen E, Veräjänkorva E. Tissue sealants may reduce haematoma and complications in face-lifts: a meta-analysis of comparative studies. J Plast Reconstr Aesthet Surg. 2017;70(3):297–306. 10.1016/j.bjps.2016.11.028. 28043785 10.1016/j.bjps.2016.11.028

[16] Gao F, Zhao Q, Zhang L, Mao X, Sun X, Zhang Y. A systematic preoperative evaluation and skin excision design for incisional blepharoplasty. J Craniofac Surg. 2024. 10.1097/SCS.0000000000010175. 39109861 10.1097/SCS.0000000000010175

[17] Wang D, Cai T, Guo F, James AJ, Yang J, Yang Y. Upper blepharoplasty through infrabrow skin excision to treat dermatochalasis with lateral hooding and medial orbital fat loss in selected patients. Ann Plast Surg. 2024;93(2S Suppl 1):S98–102. 10.1097/SAP.0000000000004006. 38896863 10.1097/SAP.0000000000004006

[18] Dell’Avanzato R, Agnelli B, Cambiaso-Daniel J, Gatti J, Gualdi A. Can local infiltration influence postoperative recovery in upper blepharoplasty? a case series study on two different infiltration methods. Facial Plast Surg. 2024;40(1):101–5. 10.1055/a-2098-6188. 37225141 10.1055/a-2098-6188

